# Anesthesia during deployment of a military forward surgical unit in low income countries: A register study of 1547 anesthesia cases

**DOI:** 10.1371/journal.pone.0223497

**Published:** 2019-10-04

**Authors:** Quentin Mathais, Ambroise Montcriol, Jean Cotte, Céline Gil, Claire Contargyris, Guillaume Lacroix, Bertrand Prunet, Julien Bordes, Eric Meaudre

**Affiliations:** 1 Department of Anesthesiology and Intensive care, Military Hospital Sainte-Anne, Toulon, France; 2 Service Médical de la Brigade des Sapeurs Pompiers de Paris, Paris, France; 3 French Military Health Service Academy Unit, Ecole du Val-De-Grâce, Paris, France; University of Colorado, UNITED STATES

## Abstract

**Background:**

Military anesthesia meets unique logistical, technical, tactical, and human constraints, but to date limited data have been published on anesthesia management during military operations.

**Objective:**

This study aimed to describe and analyze French anesthetic activity in a deployed military setting.

**Methods:**

Between October 2015 and February 2018, all patients managed by Sainte-Anne Military Hospital anesthesiologists deployed in mission were included. Anesthesia management was described and compared with the same surgical procedures in France performed by the same anesthesia team (hernia repair, lower and upper limb surgeries). Demographics, type of surgical procedure, and surgical activity were also described. The primary endpoint was to describe anesthesia management during the deployment of forward surgical teams (FST). The secondary endpoint was to compare anesthesia modalities during FST deployment with those usually used in a military teaching hospital.

**Results:**

During the study period, 1547 instances of anesthesia were performed by 11 anesthesiologists during 20 missions, totaling 1237 days of deployment in nine different theaters. The majority consisted of regional anesthesia, alone (43.5%) or associated with general anesthesia (21%). Compared with France, there was a statistically significant increase in the use of regional anesthesia in hernia repair, lower and upper limb surgeries during deployment. The majority of patients were civilians as part of medical support to populations.

**Conclusion:**

In the context of an austere environment, the use of regional anesthesia techniques predominated when possible. These results show that the training of military anesthetists must be complete, including anesthesia, intensive care, pediatrics, and regional anesthesia.

## Introduction

The French Army Health Service, a part of the French Armed Forces, ensures medical support of military personnel engaged in operations. Part of this mission includes the deployment of forward surgical teams. Their mission includes French soldiers, allies, and medical support to the populations (MSP) [1,2]. Although medical support is primarily given to military forces, the deployment of adequate means to cover most eventualities frequently leads to spare capacity to administer medical care to local populations [2]. Thus, the association of significant medical capabilities with spare capacity creates opportunities for military physicians to perform daily MSP without jeopardizing their primary purpose [3]. The activity of surgical teams has been well described in the literature, especially in Iraq, Afghanistan, and African countries [4–15]. Several publications from the surgical facilities of nongovernmental organizations (NGOs) also report their activities [16–18]. However, although all of these publications focus on the types of surgeries performed and the characteristics of the population, very few publications focus on anesthesia management [18,19].

To our knowledge, anesthesia management during a military operation has never been described in the literature. It meets unique logistical, technical, tactical, and human constraints that demand flexibility from deployed anesthesiologists and require specific anesthetic adaptations compared to standard hospital care [18,20,21]. This retrospective study reports the experience of a team of French military anesthesiologists in deployed military settings during 30 consecutive months.

## Materials and methods

### Study objectives

The principal aim of this study was to describe the anesthetic procedures performed by anesthesiologists assigned to forward surgical teams deployed in operation during the study. Secondary objectives were to describe the types of surgeries conducted, the overall impact of MSP on FST activity, transfusion requirement, and to compare anesthesia management for a few surgery types between France and missions.

### Study design

A retrospective study was performed over a period of 30 months (October 2015 to February 2018), based on an activity register. All of the anesthesiologists deployed in operation who participated in this study work at Sainte-Anne Military Hospital (SAMH). During their deployment, they had been asked to complete an activity register. This study was approved by both the SAMH ethics committee and the French Society of Anesthesiology and Intensive Care ethics committee, Paris, France (Chairperson Prof JE BAZIN) on 22 March 2018 (n° IRB 00010254-2018-021).

### Setting

French procedures of medical support in military operations follow NATO recommendations [22]. They define four respective levels of care: role 1 (immediate lifesaving non-surgical measures), 2 (initial damage control surgery before evacuation), 3 (larger facilities with more personnel), and 4 (definitive care of patients in the country of origin). Patients included in this study were in role 2 facilities. Role 2 usually includes 3 physicians (1 anesthesiologist, 1 general surgeon, and 1 orthopedic surgeon), 2 nurse anesthetists, 1 operating room nurse, 2 registered nurses, 1 radiologic technologist, 3 auxiliary nurses, and 1 administrative officer. Role 2 facilities included in this study were permanent infrastructures (Mali, Djibouti, Chad, Ivory Coast), or temporary infrastructures composed of air-conditioned tents (desert deployment) or based on the French aircraft carrier *Charles-de-Gaulle*. Role 2 facilities are organized into several subunits: an intensive care unit, a hospitalization unit, and an operating room. Two different patient paths are distinguished: elective surgery and emergencies; both include pre-operative surgical and anesthesia consultation, surgery, postanesthesia care unit or hospitalization, and discharge. Dedicated equipment for anesthesiology includes a limited choice of drugs for anesthesia, critical care, and pain management: intravenous hypnotics (propofol, gamma-hydroxybutyric acid, ketamine, midazolam, and thiopental), opioid analgesic agents (sufentanil and morphine), neuromuscular blocking drugs (succinylcholine, atracurium, and rocuronium), local anesthetics (hyperbaric bupivacaine, ropivacaine, 1% or 2% lidocaine with or without adrenaline), as well as antiemetics, reversal agents, anti-inflammatory drugs, and rescue medicines. Our pharmacy includes all of the drugs described in the 2013 *World Health Organization Model List of Essential Medicines* [23].

The standard ventilator in the initial endowment is the Elisée 350 (SAIME, Savigny-Le-Temple, France); however, a Fabius Tiro (DRAEGER, Lübeck, Germany) ventilator with an anesthetic vaporizer (sevoflurane) can be deployed in an additional lot. An M-Turbo ultrasound system is also available to perform regional anesthesia, ultrasound-guided vascular access, or point-of-care ultrasound in trauma situations. Oxygen is provided by oxygen cylinders and by SeQual Integra 10-OM (SeQual, San Diego, CA) oxygen concentrators. A peripheral nerve stimulator is also available to assist the implementation of peripheral nerve blocks. Dedicated equipment for surgery, a mobile digital radiography system, a laboratory (allowing blood analysis, such as hemoglobin and platelet count, electrolytes, HIV and hepatitis assays, and blood type testing), a surgical instrument sterilizer (2 Matachana M30-B–Matachana, Barcelona, Spain), and a blood bank with red blood cells and French lyophilized plasma packs complete the initial endowment.

Standard general anesthesia includes an intravenous induction, mainly with propofol and sufentanil. Neuromuscular blocking drug use is not mandatory and is done only if needed by the surgeon or to facilitate ventilation or intubation. Maintenance of anesthesia is performed by continuous intravenous infusion of propofol or, if an anesthesia ventilator with anesthetic vaporizer is available, by continuous inhalation of sevoflurane. Spinal anesthesia is performed with hyperbaric bupivacaine possibly associated with sufentanil. Peripheral nerve blocks are ultrasound-guided, possibly with the help of a stimulator to better identify nervous structures. Postoperative analgesia is accomplished using level 1 and 2 painkillers (paracetamol, nefopam, tramadol). Morphine titration can be performed if needed in the postanesthesia care unit.

SAMH is a 340-bed, Level 1 trauma center in the Var, France. Its anesthesiology and intensive care unit department includes 20 physicians (13 in anesthesiology and 7 in the intensive care unit, intermediate care unit, and burn center), 13 operating rooms (with thoracic, vascular, orthopedic, otorhinolaryngologic and general surgeons; neurosurgeons; and ophthalmologists; as well as a burn center and an interventional radiology suite). SAMH is the referral hospital in the province for burn patients, major trauma patients, and stroke patients.

### Population

The study included all adult or pediatric (<18 years old) patients undergoing elective or emergency surgeries during the period of deployment, with the following exceptions. Patients who received only local anesthesia were excluded. Patients treated during special operations and personnel under control were also excluded.

Data extracted from the register included demographic (sex, age, civilian or military status, etc.), surgical (type of surgery, elective or urgent surgery, etc.) and anesthesiologist data (modalities of anesthesia and postoperative analgesia, transfusion, etc.).

In 2015, the World Health Organization defined a list of essential surgeries in low-income countries [24,25]. We used this list to assess the number of performed surgeries defined as essential.

To assess the impact of the mission on anesthesia modalities, we compared anesthesia management in mission with the anesthesia management employed for a few surgeries at SAMH in France, particularly those for which either general or regional anesthesia were possible. We chose 3 subgroups of surgeries: inguinal hernia repair (open surgery procedures only), lower limb orthopedic surgery (including foot and leg surgeries for fractures), and hand and upper limb surgery (including hand, wrist, and forearm surgeries for fractures). All major patients who underwent these surgeries in SAMH in France during the period of deployment were included, and surgical and anesthesia data were compared.

### Statistical analysis

Data was collected using Excel 2010. Statistical analysis was performed with XLSTAT version 2017.18.07 (Addinsoft). Qualitative data were expressed as absolute value and percentage; quantitative data were expressed as the mean ± standard deviation or the median and interquartile range [25^th^– 75^th^ percentile].

Fisher’s exact test was used to compare anesthesia procedures between civilians and combat casualties or MSP patients in mission. We performed a multiple logistic regression analysis to evaluate the relationships of age, ASA class, sex, and anesthesia procedure with mission.

## Results

### Patient inclusion

Between October 2015 and February 2018, 20 missions were realized, totaling 1237 days of deployment in nine different theaters (five different countries). Eleven anesthesiologists were deployed for a total of 40 to 222 days of deployment per anesthetist. During the study, 1547 surgeries were completed.

### Demography

Patients’ characteristics are presented in Table 1. The majority of patients were local civilians who benefited from MSP.

**Table 1:** Demographic characteristics of patients.

	All patients (n = 1547)	Adults (n = 1282)	Pediatric patients (n = 265)
**Median age (IQR), y**	31 (22–46)	35 (27–50)	9 (5–12)
**Male, n (%)**	1154 (74.6)	988 (77.1)	166 (62.6)
**MSP, n (%)**	1318 (85)	1068 (84)	250 (94)
**French military, n (%)**	104 (7)	104 (8)	0 (0)
**Allied military, n (%)**	94 (6)	94 (7)	0 (0)
**Other, n (%)**	31 (2)	16 (1)	15 (6)
**Median weight (IQR), kg**	65 (50–74)	67 (60–75)	20 (16–31)
**Emergency surgery, n (%)**	362 (23.4)	305 (23.8)	57 (21.5)
**ASA class 1, n (%)**	1115 (72.1)	922 (71.9)	193 (72.8)

ASA, American Society of Anesthesiologists; MSP, Medical support to populations.

### Geographic distribution

**Fig 1:**
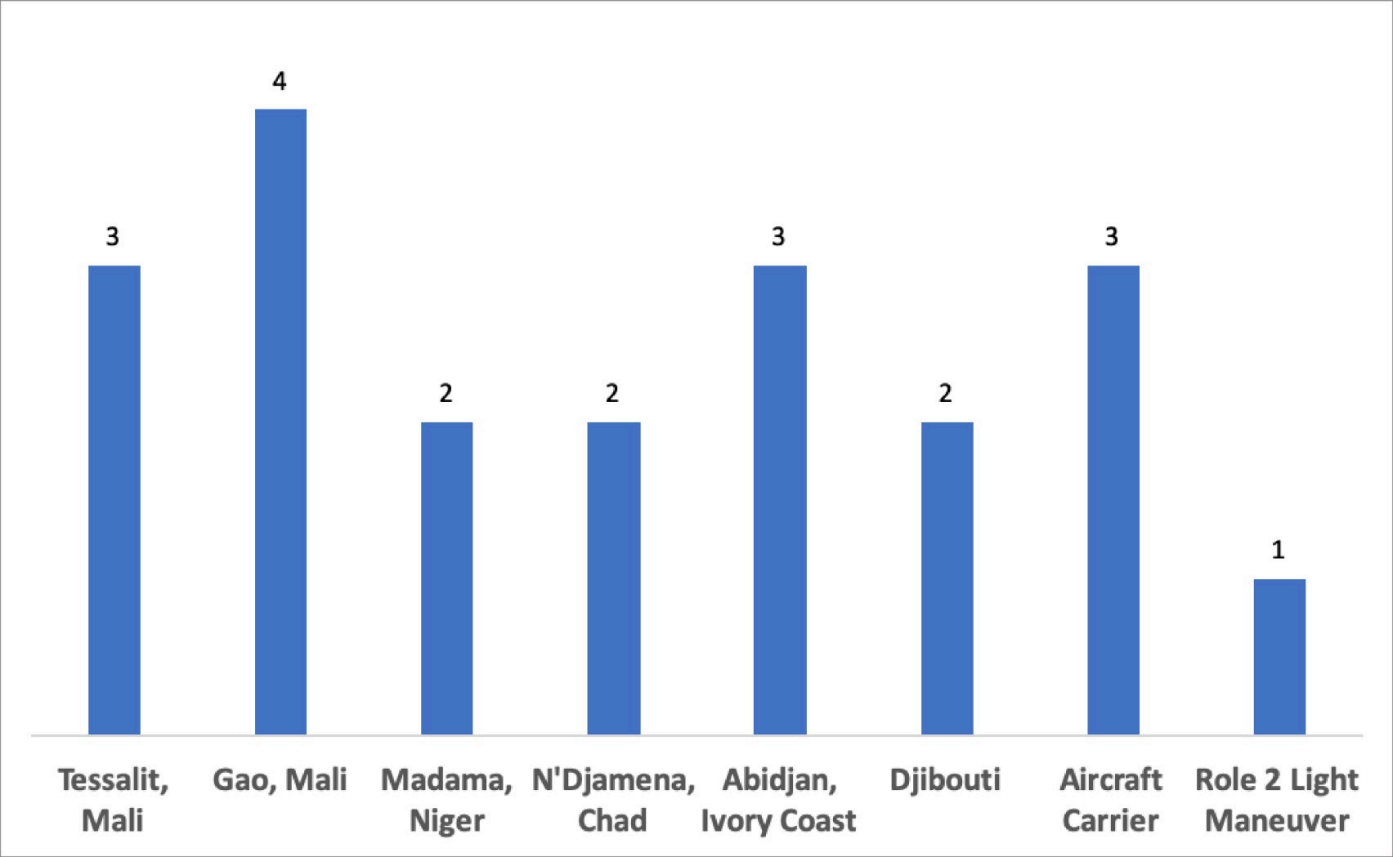
Geographic distribution of deployed forward surgical teams (FSTs) (n).

The distribution between different FSTs is represented in Fig 1. The majority of missions were conducted as part of the anti-insurgent Operation Barkhane in the Sahel-Saharan region.

### Anesthetic activity

Anesthesia was used in 1547 surgeries, divided between general anesthesia alone (549 cases, 35.5%), regional anesthesia alone (673 cases, 43.5%), and general anesthesia associated with regional anesthesia (325 cases, 21%). Anesthetic activity is detailed in Tables 2–4.

**Table 2:** Modalities of all anesthesia.

All anesthesia (n = 1547)		
	Adult (n = 1282)	Pediatric (n = 265)
**Anesthesia management**	**n (%)**	**n (%)**
General anesthesia alone	394 (30.7)	155 (58.5)
Regional anesthesia alone	649 (50.6)	24 (9.1)
**Major Complications**	**n (%)**	**n (%)**
Shock–postoperative vasopressors	27 (2.1)	1 (0.4)
Profound desaturation (SpO_2_<80%)	10 (0.8)	5 (1.9)
Difficult intubation	4 (0.3)	0 (0)
Laryngospasm	0 (0)	1 (0.4)
Anaphylactic shock	1 (0.1)	0 (0)
Total spinal anesthesia	1 (0.1)	0 (0)
Breach during epidural anesthesia	1 (0.1)	0 (0)
Dental damage	1 (0.1)	0 (0)

**Table 3:** Modalities of regional anesthesia.

Regional anesthesia (n = 1224)		
	Adult (n = 1115)	Pediatric (n = 109)
Type of regional anesthesia	n (%)	n (%)
Spinal anesthesia	527 (47.3)	12 (11.2)
Transversus abdominis plane (TAP) block	258 (23.1)	37 (34.6)
Axillary brachial plexus block	91 (8.2)	11 (10.3)
Femoral nerve block	70 (6.3)	6 (5.6)
Sciatic nerve block	48 (4.3)	12 (11.2)
Interscalene brachial plexus block	25 (2.2)	10 (9.3)
Epidural anesthesia	34 (3.0)	0 (0)
Fascia iliaca block	20 (1.8)	2 (1.9)
Parietal infiltration	12 (1.4)	5 (4.7)
Ilioinguinal block	10 (0.9)	2 (1.9)
Pudendal nerve block	10 (0.9)	0 (0)
Ankle block	1 (0.1)	4 (3.7)
Hand block	3 (0.3)	0 (0)
Saphenous block	1 (0.1)	0 (0)
Pectoralis (PEC) block	1 (0.1)	0 (0)
Other	0 (0)	8 (7.5)
**Local anesthetic**	**n (%)**	**n (%)**
Ropivacaine (peripheral block)	633 (95.5)	93 (95.9)
Lidocaine (peripheral block)	30 (4.5)	4 (4.1)
Bupivacaine hyperbaric (spinal anesthesia)	527 (100)	12 (100)
**Regional anesthesia success**	**n (%)**	**n (%)**
Success	1089 (97.7)	109 (100)
Failure	26 (2.3)	0 (0)

**Table 4:** Modalities of general anesthesia.

General anesthesia (n = 873)		
	Adult (n = 633)	Pediatric (n = 240)
**Airway access**	**n (%)**	**n (%)**
Orotracheal intubation	625 (71.5)	131 (58.5)
General anesthesia with spontaneous ventilation	203 (23.2)	81 (36.2)
Laryngeal mask airway	46 (5.3)	12 (5.3)
**Neuromuscular blocking drugs (NMB)**	**n (%)**	**n (%)**
No NMB	474 (74.9)	229 (95.4)
NMB	159 (25.1)	11 (4.6)
Vecuronium	65 (35.7)	1 (9.1)
Succinylcholine	55 (30.2)	9 (81.8)
Atracurium	38 (20.9)	1 (0.1)
Rocuronium	24 (13.2)	0 (0)
**Induction of anesthesia**	**n (%)**	**n (%)**
Propofol	445 (80.6)	90 (45.7)
Sevoflurane	2 (0.4)	104 (52.8)
Ketamine	69 (12.5)	3 (1.5)
Etomidate	18 (3.3)	0 (0)
Already under general anesthesia	18 (3.3)	0 (0)
**Maintenance of anesthesia**	**n (%)**	**n (%)**
Sevoflurane	353 (91.9)	171 (97.1)
Propofol	23 (6)	4 (2.3)
Midazolam	5 (1.3)	1 (0.6)
Ketamine	3 (0.8)	0 (0)
**Mean arterial pressure (MAP)**	**Mean ± SD**	**Mean ± SD**
Preoperative MAP	92.5 ± 13.7	77.0 ± 12.7
Lowest peroperative MAP	73.6 ± 12.7	64.8 ± 12.9
Postoperative MAP	86 ± 11.7	76.6 ± 13.0

Regional anesthesia was categorized as perimedullar anesthesia (spinal anesthesia and epidural anesthesia) or peripheral regional anesthesia (eg, axillary brachial plexus block, femoral nerve block, sciatic nerve block). The majority of perimedullar blocks were spinal anesthesia (539 cases, 34.8%).

Multiple types of peripheral blocks were used. We identified 295 transversus abdominis plane (TAP) blocks (19%), 102 axillary brachial plexus blocks (6.6%), 76 femoral nerve blocks (4.9%), 60 sciatic nerve blocks (3.9%), and 118 other types of peripheral block (eg, fascia iliaca block, pudendal nerve block, ankle block; 7.6%). The majority of the peripheral blocks were ultrasound guided (89.4%). Twenty-eight patients (1.8%) required an immediate postoperative morphine titration.

In all, 1218 patients (78.7%) were hospitalized in the ward for ≥1 day after surgery; 275 patients (17.8%) benefited from an ambulatory surgery. Fifty-four patients (3.5%) were hospitalized in the intensive care unit after surgery. Two patients (0.13%) died during surgery.

### Comparison with France

**Fig 2:**
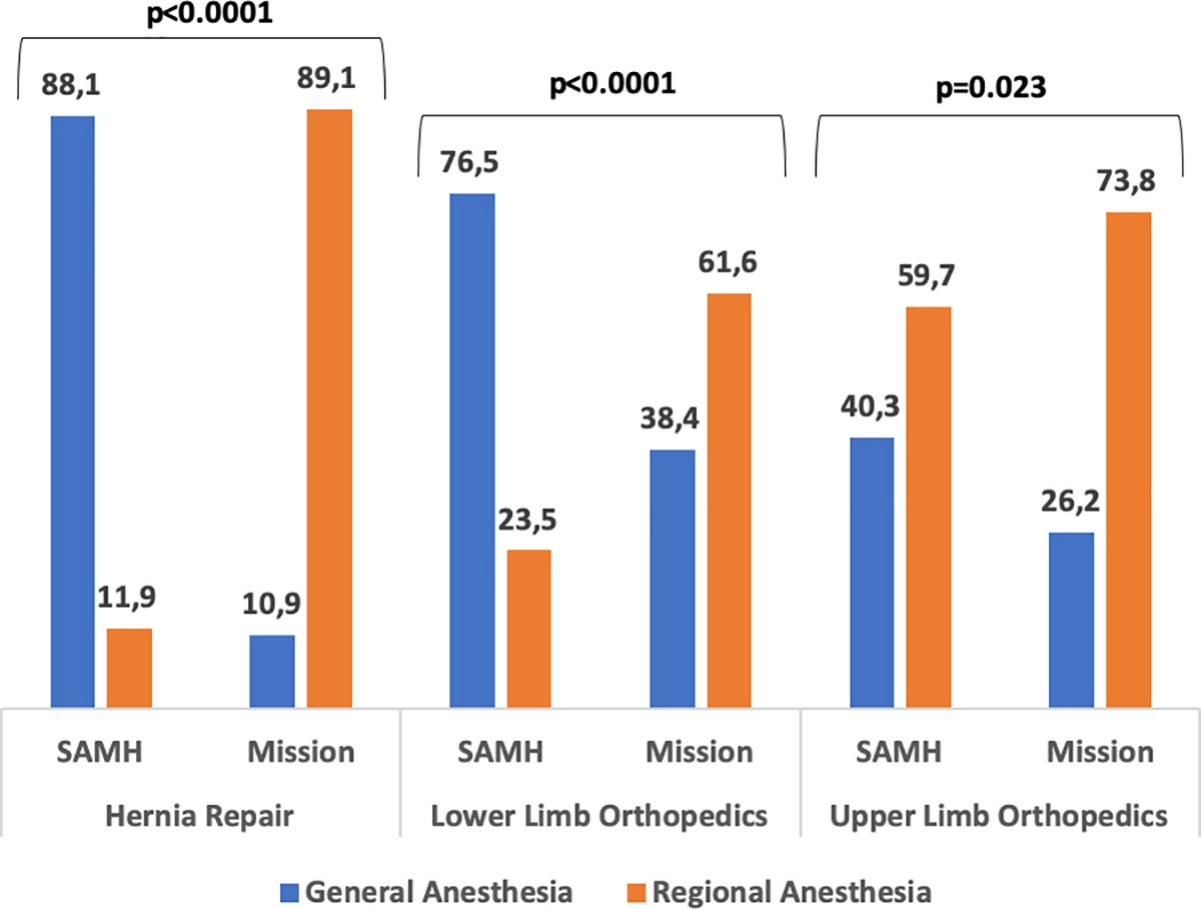
Anesthetic management in France and in mission. SAMH: Sainte Anne Military Hospital.

During the study, 22 979 adult patients were screened in SAMH in France. We identified 105 patients in the open surgery hernia repair subgroup, 405 patients in the lower limb orthopedic surgery subgroup, and 408 in the hand and upper limb surgery subgroup. During the same period in mission, we included 304 adult patients in the open surgery hernia repair subgroup, 112 patients in the lower limb orthopedic surgery subgroup, and 80 in the hand and upper limb surgery subgroup. There was a statistically significant increase in the use of regional anesthesia in mission versus French hospital for all groups (Fig 2 and Table 5).

**Table 5:** Factors associated with anesthesia in missions compared to a French hospital.

Variable	Odds Ratio	p value
Hernia repair		
Regional Anesthesia	78.4 (42.5–155)	<0.001
Age	0.94 (0.92–0.96)	<0.001
ASA class ≥2	0.36 (0.18–0.67)	<0.01
Male	0.56 (0.22–1.4)	0.22
Upper limb orthopedics		
Regional Anesthesia	1.9 (1.1–3.6)	0.028
Age	0.97 (0.95–0.99)	<0.01
ASA class ≥2	0.16 (0.1–0.34)	<0.001
Male	1.6 (0.8–3.3)	0.19
Lower limb orthopedics		
Regional Anesthesia	5.6 (3.4–9.6)	<0.001
Age	0.97 (0.95–0.99)	<0.001
ASA class ≥2	0.235 (0.13–0.42)	<0.001
Male	2.57 (1.24–5.17)	<0.01

Multivariate model adjusted for sex, age, ASA classification and type of anesthesia.

ASA, American Society of Anesthesiologists.

### Surgical activity

The distribution of different surgical specialties is presented in Table 6.

**Table 6:** Types of surgery.

Surgery type	Adults (n = 1355)	Pediatrics (n = 296)
	**n (%)**	**n (%)**
Orthopedic	409 (31.9)	83 (31.3)
Abdominal	396 (30.9)	62(23.4)
Soft tissues	192 (15)	32 (12.1)
Otorhinolaryngology	93 (7.3)	10 (3.8)
Urology	92 (7.2)	22 (8.3)
Gynecology	70 (5.5)	2 (0.8)
Wound treatment	62 (4.8)	38 (14.3)
Thoracic	19 (1.5)	2 (0.8)
Neurosurgery	13 (1)	1 (0.4)
Burn surgery	9 (0.7)	44 (16.6)

The majority of surgeries were elective and part of MSP (1185 cases, 76.6%); 362 (23.4%) were emergency cases. Nearly two-thirds (63.3%) of the surgeries performed on MSP patients were defined as essential by the WHO (Table 7).

**Table 7:** WHO essential surgeries in MSP patients.

Procedure type	n (%)
Hernia	368 (27.9)
Drainage of superficial abscess	28 (2.1)
Appendectomy	10 (0.8)
Gallbladder disease	6 (0.5)
Hydrocelectomy	50 (3.8)
Relief of urinary obstruction	18 (1.4)
Fracture treatment	318 (24.1)
Amputation	22 (1.7)
Tube thoracostomy	4 (0.3)
Skin grafting	10 (0.8)
**Total MSP surgeries**	**1318 (100)**
**WHO essential surgeries**	**834 (63.3)**

MSP, Medical support to populations; WHO, World Health Organization.

Emergency surgeries concerned 185 civilians in relation with MSP (51.1%), 99 French military personnel (27.4%), 71 allied military personnel (19.6%), and 7 soldiers from other military forces (1.9%).

In all, 138 surgeries were trauma-related, concerning 52 civilians in relation with MSP (37.7%), 51 allied military personnel (37%), 29 French military personnel (21%), and 6 soldiers from other military forces (4.3%). The origins of trauma were multiple and included the following: ballistic trauma (49 cases, 35.5%); motor vehicle collision (33 cases, 23.9%), shrapnel wounds (22 cases, 15.9%), and diverse causes (eg, sports injuries, brawls) (30 cases, 21.7%).

### Build operational efficiency

For all of the anesthesiologists deployed, elective surgery on MSP patients began 1 to 3 days after their arrival. Median time before the first war-related wounded soldier or mass-casualty influx was 10 days (3–24), representing 7 (2–33) patients.

### Transfusions

During the study period, 70 patients (4.5%) received blood products. In all, 74 units of packed red blood cells (RBCs) were transfused into 35 patients, and 46 units of French lyophilized plasma (FLYP) were transfused into 21 patients. Eleven patients received fibrinogen concentrate, 28 patients received an auto transfusion, and 9 patients received a transfusion of fresh whole blood.

## Discussion

To our knowledge, this study is the first to describe anesthesia techniques and modalities provided by military anesthesiologists during their deployments. It provides insight into the types of anesthesia services needed in mission and highlights several issues specific to anesthesia management in austere environments. Despite the material and human resources available in French role 2 facilities, which make it possible to perform quality anesthesia safely as in France, the austere environment specific to war zones and the need to preserve medical operational readiness capabilities for injured soldiers influences physicians’ choices concerning anesthesia modalities [21]. Physical, human, logistical, and tactical imperatives specific to austere environments require great flexibility and adaptability in the practice of anesthesia.

### Regional anesthesia in austere environments

First, regional anesthesia has a prominent place in forward surgical teams, particularly for elective surgery. Whenever possible, surgeries are performed under regional anesthesia, either alone or in combination with general anesthesia. For some surgeries performed in France (open surgery hernia repair; lower limb orthopedic surgery; hand and upper limb orthopedic surgery), anesthesiologists deployed in mission preferentially chose regional anesthesia. Regional anesthesia in austere environments has several advantages: low or no need for perioperative oxygen; good postoperative analgesia, especially among ambulatory surgery patients; and early discharge from the postoperative recovery room and hospital [26–28]. Neuraxial anesthesia, particularly spinal anesthesia, is frequently used for the majority of lower limb orthopedic surgeries, and also for urologic and visceral surgeries [26]. It is a simple, effective, and low-cost procedure allows early discharge and provides good analgesia. Epidural anesthesia was less frequently chosen because it requires more intensive postoperative monitoring, which is not possible in wards in austere environments. Peripheral nerve blocks are also very useful alone, especially for upper limb orthopedic surgeries, and are also paired with general or spinal anesthesia to ensure optimal postoperative analgesia. The availability of an ultrasound machine in the initial endowment of the forward surgical teams may facilitate the realization of these blocks and may also shorten performance time and the onset time [29,30]. During painful abdominal surgeries, combination of ultrasound-guided peripheral nerve blocks (such as Transversus Abdominis Plane block) with general anesthesia provides an effective alternative to epidural analgesia [31]. In the literature, the main study focusing on anesthesia in low and middle income countries is a large cohort study describing Médecins Sans Frontières activity during 6 years [18]. Regional anesthesia (and particularly spinal anesthesia) also had an important place: 52% of surgeries were conducted under regional anesthesia, including 46% of spinal anesthesia. However, most of the surgeries completed were emergent or time sensitive, with a very different repartition of surgical specialties (45% of gynecologic/obstetric and urologic surgeries).

### Place of medical support to populations

Second, in addition to the medical support provided to French and allied military forces, FSTs also provide MSP. The French army has a long tradition of political and military presence in Francophone Africa, and MSP has always been a part of its action. MSP presents several benefits: help civilians who need emergent or elective surgery in low-income countries with very limited medical facilities; “win heart and minds” of local citizens and preserve good relations with local nations; build operational efficiency; and maintain good team cohesion (members of mixed medical and paramedical teams coming from different hospitals in France do not always know each other beforehand) [32]. However, FSTs should be aware of different pitfalls that accompany MSP practice, and prevent them [15]. MSP must continue to work with the local health system. FSTs should avoid the treatment of chronic problems because follow-up of patients can be hazardous and material resources are limited. Surgeons should focus on pathologies that can be cured with one surgery and require at best no follow-up, as described in WHO’s list of essential surgeries [14,15,23,32–34]. Finally, MSP remains a secondary purpose in French army doctrine, and must not interfere with the care of wounded soldiers. Anesthesiologists and surgeons must save their resources while performing MSP and anticipate a possible mass casualty incident; hence the advantages of regional anesthesia allow early discharge for patients without risks or pain, as well as economical use of medical equipment. Given these conditions, the risk-benefit balance of MSP seems to remain favorable.

### Blood, oxygen and emergencies management

In operations, the French Army and Health Service provides important means to the surgical teams to cover most eventualities. Spare capacity is often available to perform MSP [3]. However, three concerns must be raised and anticipated in this context: transfusion, oxygen, and the management of surgical emergencies among the civilians. Concerning blood management, role 2 blood banks are not extensive, and potentially hemorrhagic surgeries should be limited as much as possible. Concerning oxygen management, preoxygenation and oxygenation in the postoperative room can be done using oxygen extractors [35]. However, general anesthesia requires high-pressure oxygen flow from a cylinder to the Fabius Tiro ventilator. To save oxygen for wounded soldiers requiring general anesthesia or high FIO2 ventilation in the intensive care unit, the best solution is to maximize the use of regional anesthesia. Finally, postoperative facilities, and especially intensive care units, are not comprehensive. Emergencies tend to saturate the postoperative ward or the intensive care unit, and anesthesiologists must be careful about whether or not they accept emergency patients. Military and NGO MSP differ in their management of emergencies and logistics, so it is difficult to compare them. It may yet lead to an ethical dilemma for military medical teams in the event that care of a patient might not be possible (eg, severely burned patients, surgical oncology) [36–40].

Among MSP patients, we found an important sex disparity, as a large majority of men (72.8%) have benefited from surgical management during our study. However, there is a strong male preponderance in both conflict-related and non-conflict-related trauma worldwide that may explain this disparity, at least in part [41]. Yet, in non-trauma-related pathologies, the majority of those who received surgical care by FSTs were still men (69.1%). These results are consistent with the literature, in both civilian and military structures [14,15,41]. Several causes were mentioned: cultural or religious reasons may require that women be accompanied by men outside of the home or that women only receive care from female health care providers, or women may resort first to traditional healers [42–45]. Nevertheless, it is important to be aware of these disparities to ensure appropriate care for patients in an already tragic situation.

### Pediatric anesthesia

Finally, another interesting aspect of anesthesia in missions is the proportion of pediatric patients: 17.1% of patients in this study were younger than 18 years old, and 2.4% were younger than 2 years old or weighed <10 kg. Pediatric patients present special anatomical, physiological, and pharmaceutical considerations, as well as specific perioperative adverse events [46]. However, in France, military anesthesiologists rarely take care of children in operative rooms and pediatric management is another specificity of our work during missions.

### Limits

This study has several limitations. It is a retrospective study, although the anesthesiologists collected data during their deployment, and there may have been some omissions. It is a single-center study, as all the anesthesiologists came from a single hospital in France. However, 40% of the anesthesiologists included in this study were trained at another military hospital in France before their transfer in Toulon. We did not perform a systematic follow up of patients, and data concerning postoperative management (eg, pain, length of stay) are missing. Finally, we excluded data from prisoners of war and special operations.

## Conclusion

This work is the first to describe the anesthesia techniques used during military deployments. In the context of an austere environment, it reveals the predominant use of regional anesthesia techniques when possible. These results show that the training of military anesthetists must be complete. It would be interesting to have studies in other armies.

## Abbreviations

SAMH: Sainte-Anne Military Hospital
MSP: Medical support to the population
FST: Forward surgical team
RBC: Red blood cell
WHO: World Health Organization
NGO: Nongovernmental organization
